# Supplementary figures and images for: The tRNA thiolation-mediated translational control is essential for plant immunity
Source: eLife. 2024 Jan 29;13:e93517. doi: 10.7554/eLife.93517 (PMC10863982; doi:10.7554/eLife.93517)

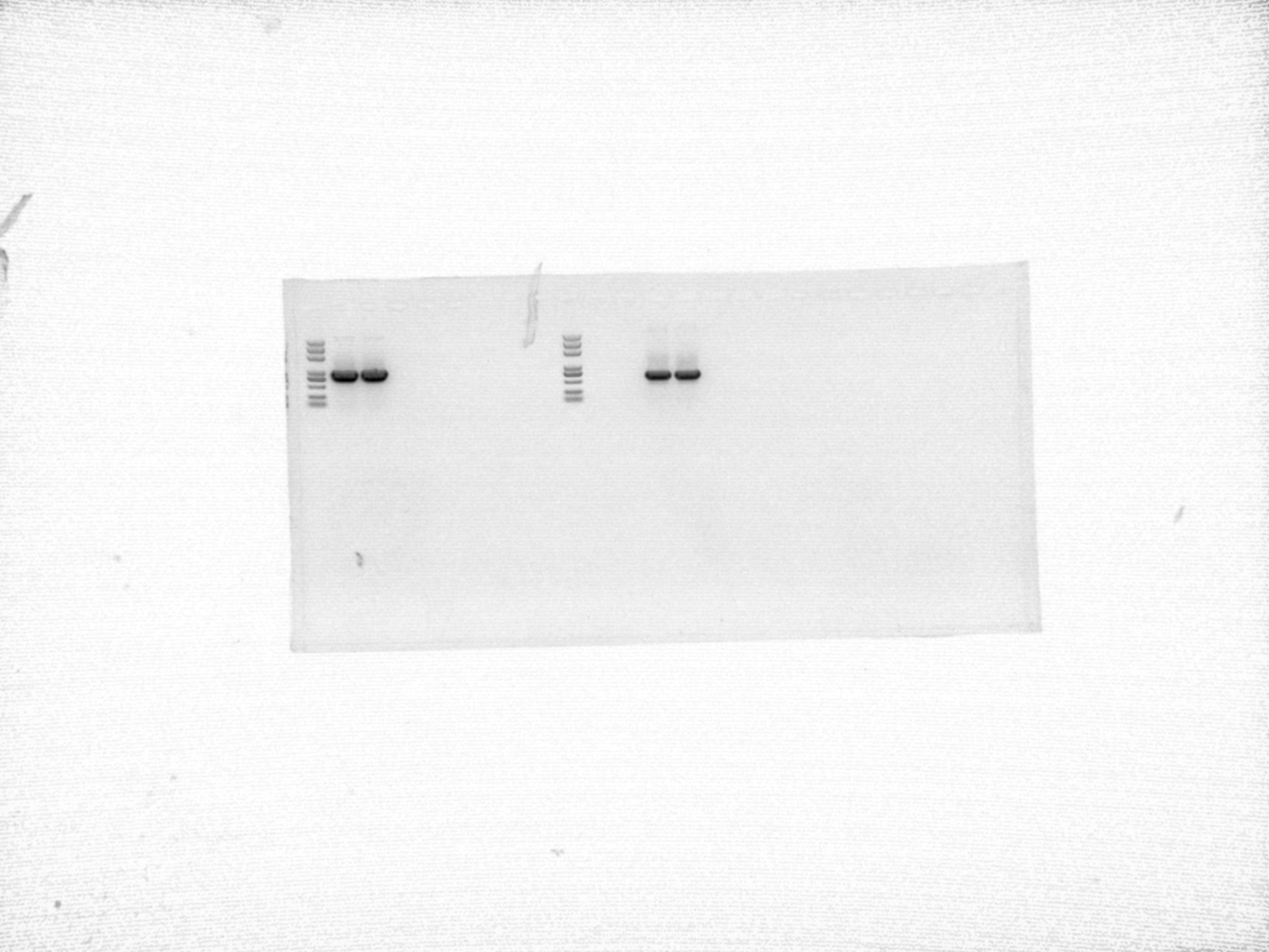

Supplement: Figure 1—source data 2. [file elife-93517-fig1-data2.zip › Figure 1-Source Data 2/Figure 1D-raw.tif]

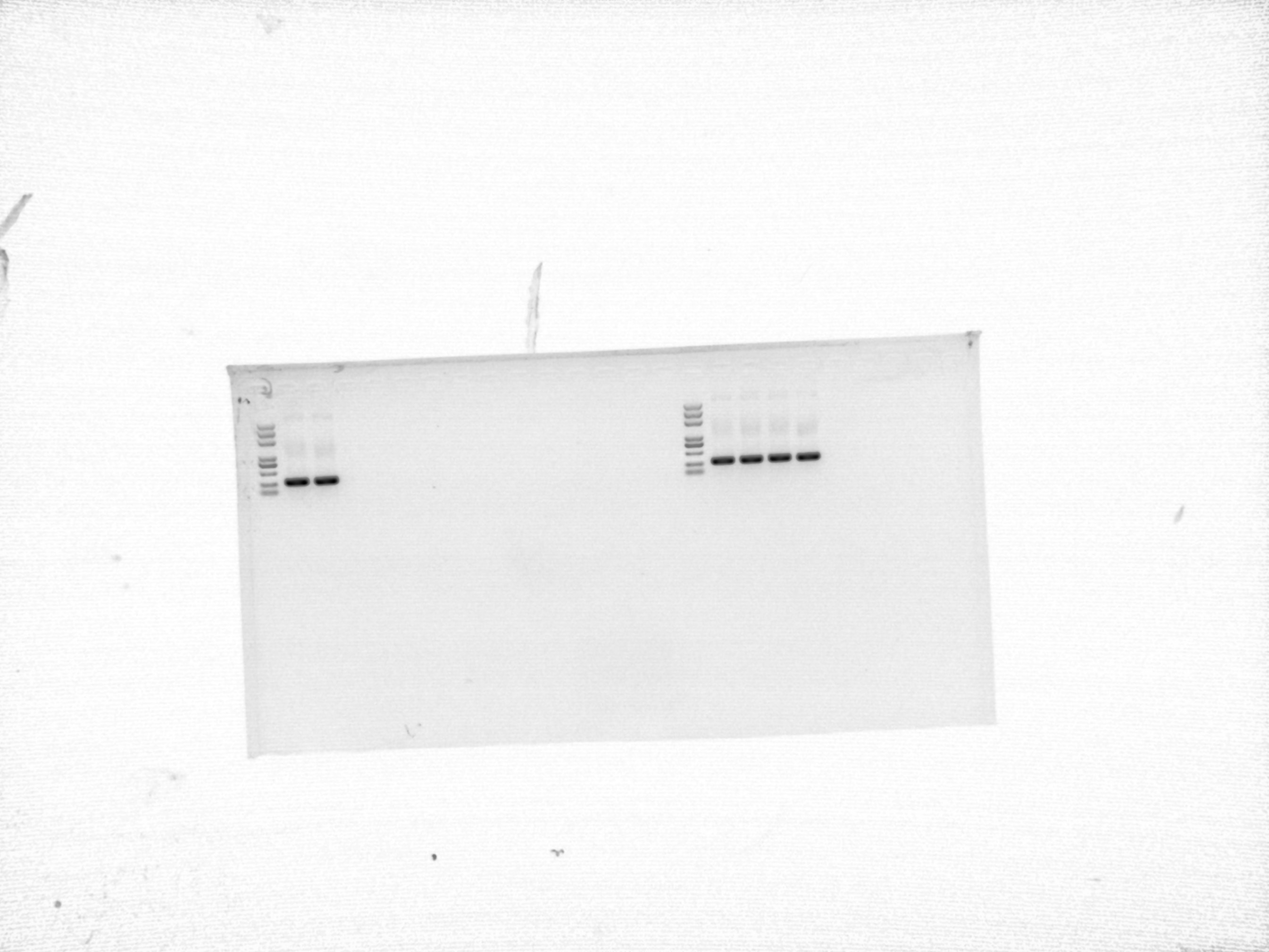

Supplement: Figure 1—source data 3. [file elife-93517-fig1-data3.zip › Figure 1-Source Data 3/Figure 1E-raw.tif]

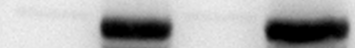

Supplement: Figure 2—source data 1. [file elife-93517-fig2-data1.zip › Figure 2-Source Data 1/Figure 2D-raw2-1.tif]

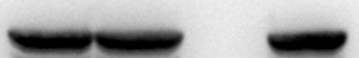

Supplement: Figure 2—source data 1. [file elife-93517-fig2-data1.zip › Figure 2-Source Data 1/Figure 2D-raw1-1.tif]

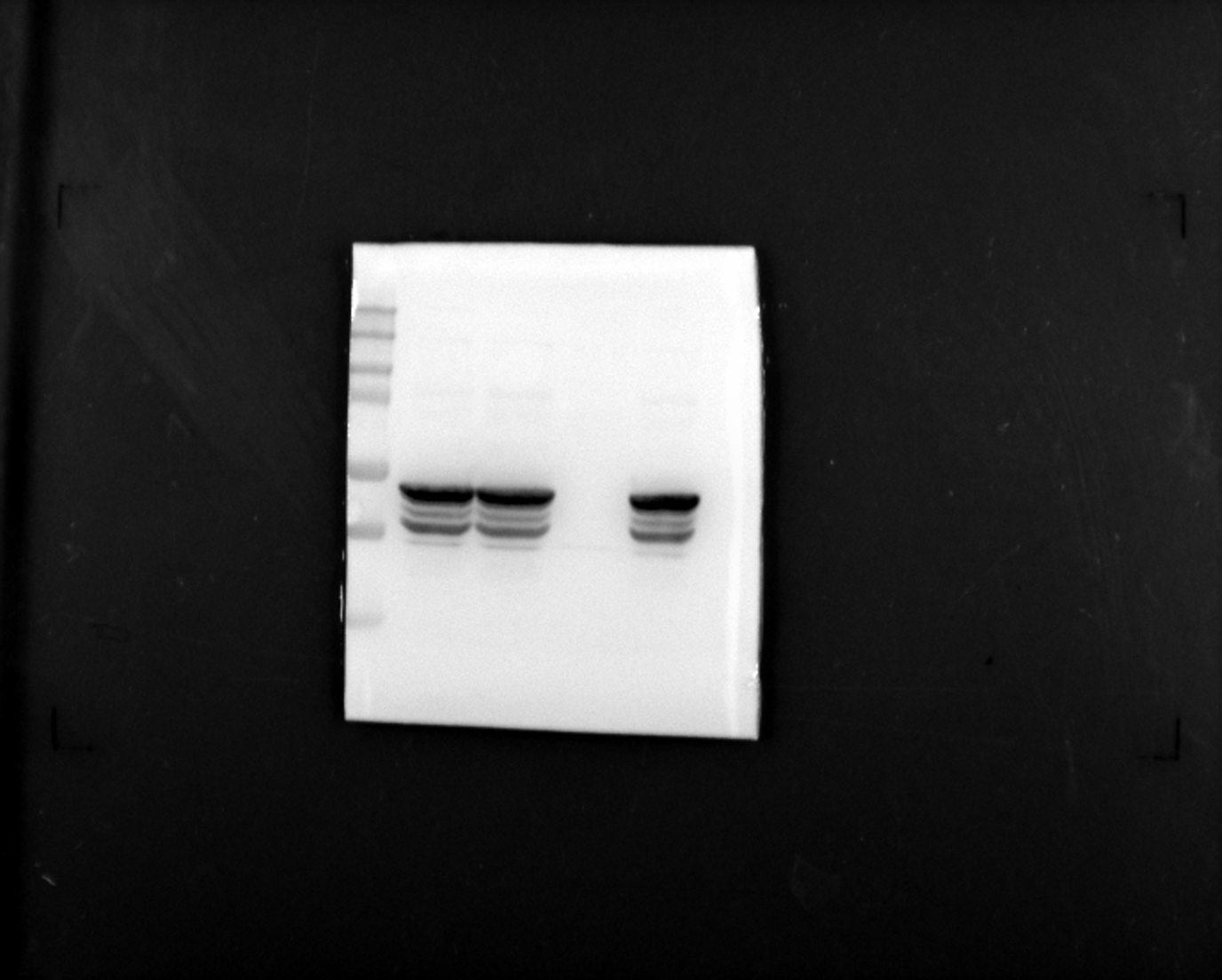

Supplement: Figure 2—source data 1. [file elife-93517-fig2-data1.zip › Figure 2-Source Data 1/Figure 2D-raw1.tif]

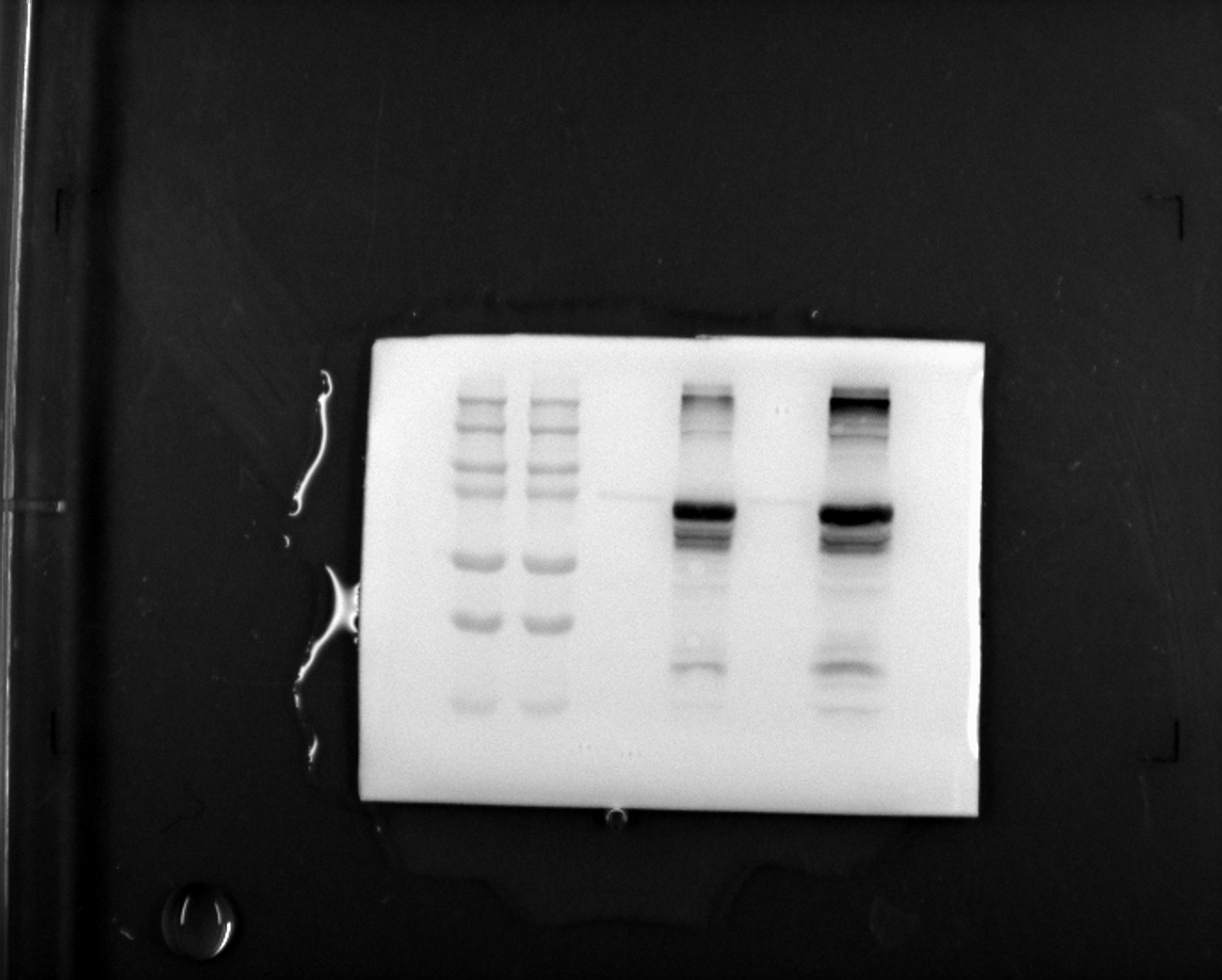

Supplement: Figure 2—source data 1. [file elife-93517-fig2-data1.zip › Figure 2-Source Data 1/Figure 2D-raw2.tif]

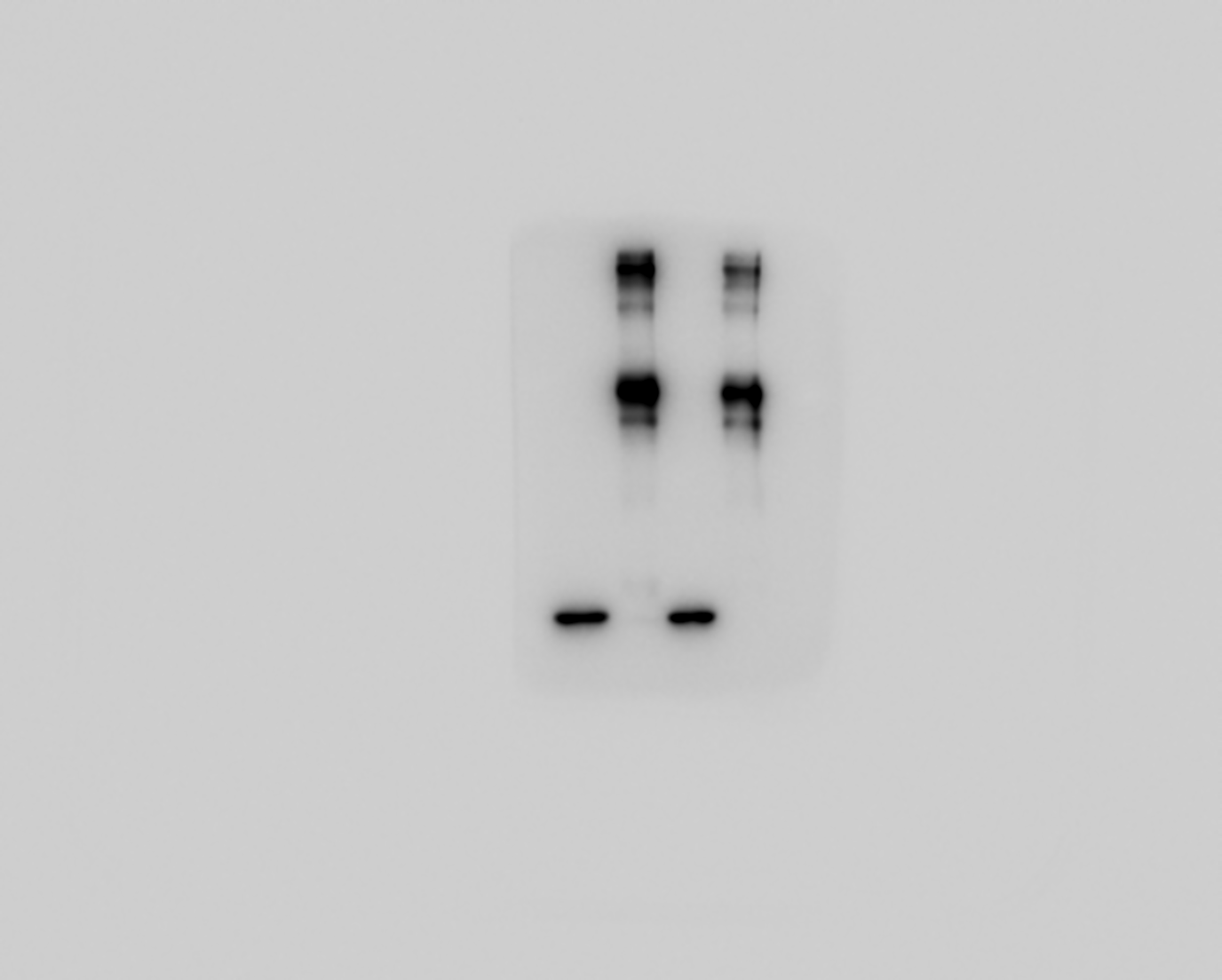

Supplement: Figure 2—source data 2. [file elife-93517-fig2-data2.zip › Figure 2-Source Data 2/Figure 2E-raw1.tif]

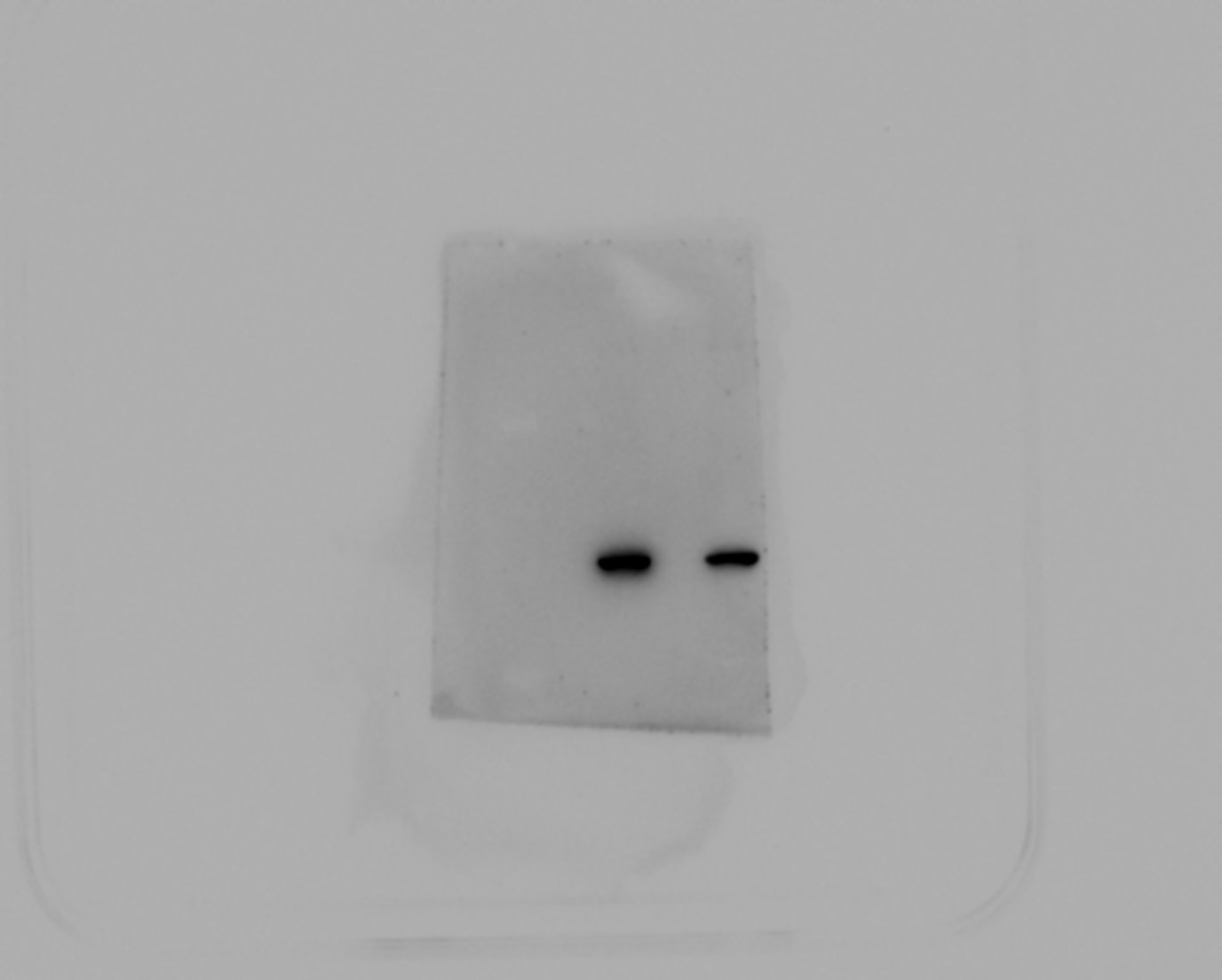

Supplement: Figure 2—source data 2. [file elife-93517-fig2-data2.zip › Figure 2-Source Data 2/Figure 2E-raw2.tif]

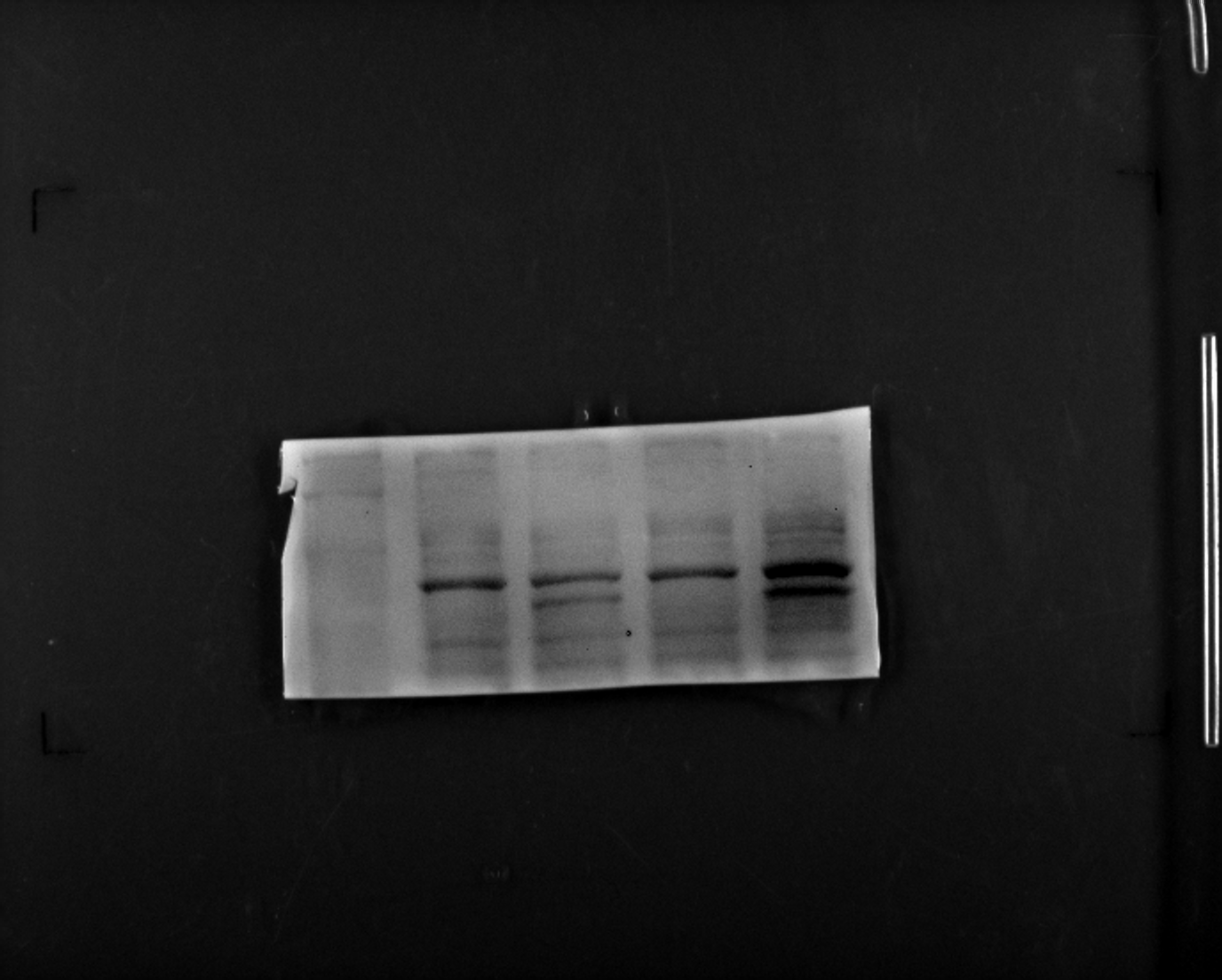

Supplement: Figure 5—source data 3. [file elife-93517-fig5-data3.zip › Figure 5-Source Data 3/Figure 5C-raw1.tif]

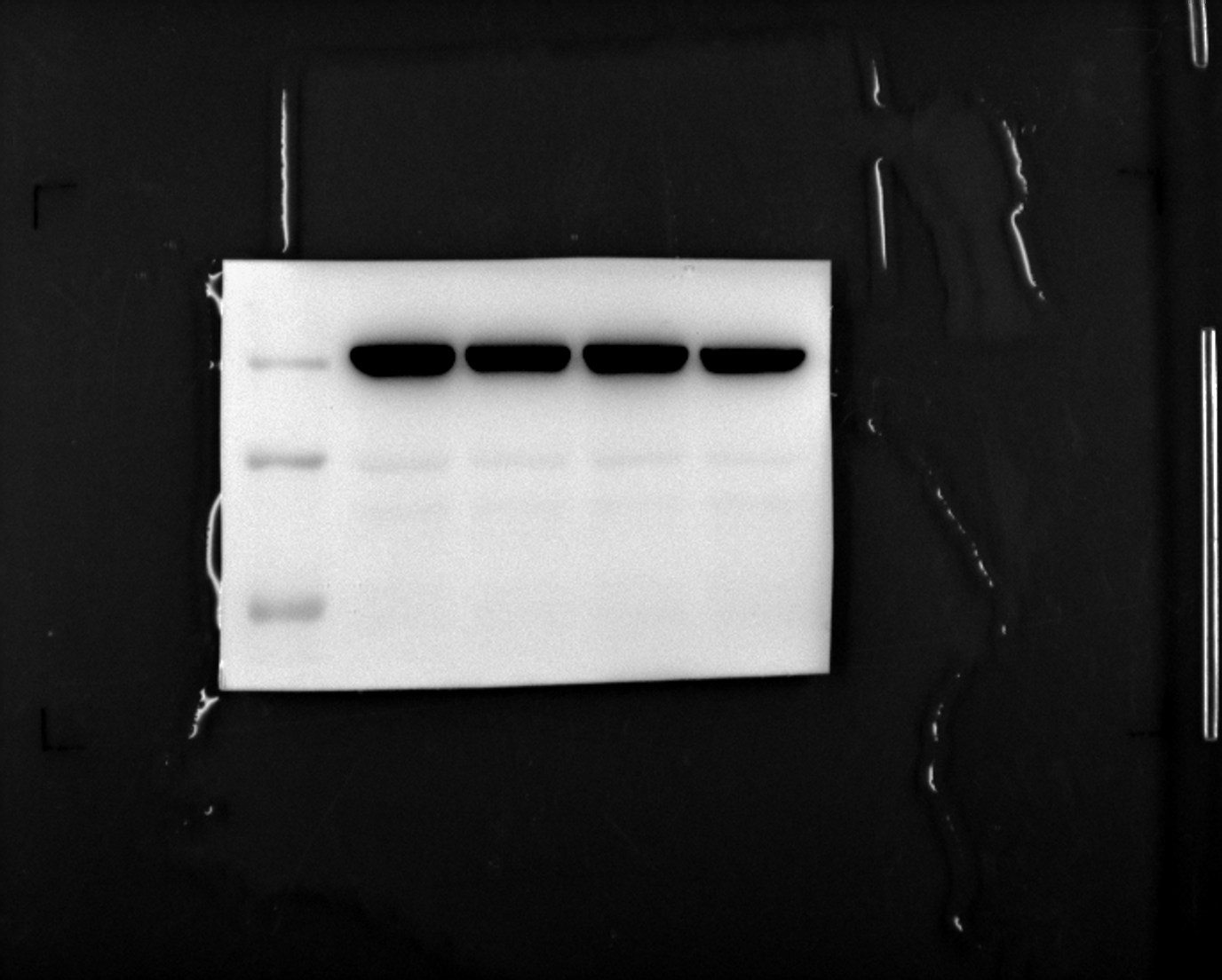

Supplement: Figure 5—source data 3. [file elife-93517-fig5-data3.zip › Figure 5-Source Data 3/Figure 5C-raw2.tif]
